# The interaction of occupational stress and job burnout on depressive symptoms in railway workers in Fuzhou city

**DOI:** 10.1186/s12889-024-18902-2

**Published:** 2024-05-29

**Authors:** Shuangjie Yu, Min Zhao, Ye Li, Can Liu, Youjuan Fu, Yu Jiang, Suzhen Guan

**Affiliations:** 1 School of Public Health, The Key Laboratory of Environmental Factors and Chronic Disease Control of Ningxia, Ningxia Medical University, Yinchuan, Ningxia, 750004 China; 2 Department of Preventive Medicine, School of Public Health, Fujian Medical University, Fuzhou, Fujian 350122 China

**Keywords:** Rail workers, Job burnout, Occupational stress, Depressive symptoms, Interaction effect

## Abstract

**Background:**

To explore the relationship between occupational stress, burnout and depressive symptoms among railroad workers in Fuzhou, and to analyze the interaction of burnout and occupational stress on depressive symptoms.

**Methods:**

In this study, 861 railway employees of Fuzhou railway bureau were randomly selected from January to April, 2022. Occupational stress inventory revised edition (OSI-R), China job burnout inventory (CMBI) and Symptom Checklist-90 (SCL-90) were used to investigate the occupational stress, job burnout and depressive symptoms of railway workers. Interactions associated with depressive symptoms were assessed by linear hierarchical regression analysis and SPSS macros (PROCESS).

**Results:**

Occupational stress, job burnout and depressive symptoms accounted for 50.58%, 93.47%, and 11.19% of the study population, respectively. There were intergroup differences between age, marriage status, and length of service (*P* < 0.05). Occupational stress and job burnout are the main risk factors for depressive symptoms (*OR*: 2.01, 95% *CI*: 1.17–3.45; 1.94, 1.69–2.23, respectively). More importantly, further analysis of the interaction between occupational stress and job burnout showed that those with high levels of job burnout had a high-risk effect on depressive symptoms at high levels of occupational stress.

**Conclusion:**

Occupational stress and job burnout are risk factors for depressive symptoms among railroad workers in Fuzhou City. The interaction of job burnout and occupational stress increases the risk of depressive symptoms.

## Background

Along with economic development, workers’ lifestyles and work environments have constantly changed with the advent of new occupational hazards. Psychosocial factors’ influences on physical and mental health have brought extensive attention internationally [1]. Occupational stress and burnout are hot issues in occupational health at home and abroad [2].

Occupational stress refers to the physical and psychological stress caused when the individual’s abilities, coping resources, and needs are not balanced with the demands of the job. It has been recognized as a significant issue affecting occupational health globally [3, 4]. Occupational stress is associated with sub-health manifestations, including coronary diseases, digestive system diseases, sleep disorders, depressive symptoms, anxiety, etc [5–7]. Studies have shown that people with high occupational stress are more likely to suffer from depressive symptoms than the general population [8]. Job burnout is delineated as a state of physical, emotional, and mental exhaustion resulting from prolonged engagement in emotionally demanding work [9]. It concerns somatic diseases and psychological disorders and can lead to depressive symptoms and even suicidal thoughts [10, 11]. It was shown that a higher level of job burnout led to severer depressive symptoms [12].

Depressive symptoms is a common mental health problem caused by occupational stress and burnout. It is also a primary mental disease endangering human health [13–15]. WHO reported that the annual prevalence rate of depressive symptoms in females and males is 9.5% and 5.8%, respectively. Patients mainly manifest prolonged low mood and helplessness [16]. Research indicated that depressive symptoms cases rose by 49.86% from 1990 to 2017 globally, and depressive symptoms patients have exceeded 300 million worldwide [17]. According to WHO’s projections, depressive symptoms will probably become the most burdensome disease worldwide in 2030 [16]. The occupational population exhibits a higher depressive symptoms tendency with rising occupational stress and burnout [18, 19]. The results of a study on the Effect of occupational stress on the mental health of railway workers showed that The detection rates of occupational stress, mental health problems and depression among workers in the railroad engineering system were 40.5% (248/613), 9.0% (55/613) and 40.78% (250/613), respectively [20]. In a previous study, the authors found that a total of 532 out of 1,270 railroad workers were identified as having mental health problems, and those experiencing high levels of occupational stress had a higher risk of poor mental health (*OR* = 1.80,95% *CI*: 1.31–2.47) [21]. In another study, the authors also found that occupational stress was one of the most important factors contributing to psychological problems among railroad drivers [22]. Moreover, it was reported that job burnout might mediate the effects of occupational stress on depression [23].

Interactions can more accurately describe and explain how different factors work together to influence an outcome, helping to reveal nonlinear and conditional relationships between variables [24]. It was demonstrated that job burnout might play a moderating role between occupational stress and depression among doctors, nurses, and teachers [25–27]. Therefore, we further explored the relationship between occupational stress, burnout, and depression to focus on the impact of the interaction of burnout and occupational stress on depressive symptoms. This is important for preventing mental health problems among railroad workers and extending the life cycle of employees.

## Methods

### Participants and procedure

The estimated expected prevalence was referred to the prevalence of adult depression in mainland China [28], *P* = 23.4%, α = 0.05, δ = 0.03, *N* = 741 cases, considering the bias to increase the sample size by 10%, the sample size to be investigated was 815 cases. Therefore, in this study, 861 workers (700 men and 158 women) who have been on duty for more than one year in Fuzhou Railway Bureau were randomly selected in January-April 2022 by using cluster sampling as the research subjects for cross-sectional study. The inclusion criteria included employees who were working for more than one year with an excellent mental state and no a history of mental illness or disorder. If participants using any medication for mental health problems in the three months prior to the study will be excluded. Participants ranged from 18 to 60 years (39.80 ± 9.16 years).

$$N=\left[\left(Z_1-\alpha / 2\right) / \delta\right]^2 \times p \times(1-p)$$

An online self-reported questionnaire was employed to collect information on demographic characteristics and lifestyle questions, accompanied by measures of occupational stress, job burnout and depressive symptoms. 861 questionnaires were distributed, and 858 valid questionnaires were returned (99.65%). Approval was granted by the Ethics Committee of Ningxia Medical University (No.2022-N079). All participants provided written informed consent.

### Occupational stress

To investigate occupational stress, stress response, and resilience among subjects, a Chinese version of the instrument called the revised edition of the occupational stress inventory (OSI-R) proposed by Oispow was used [29]. This questionnaire comprises the Occupational Roles Questionnaire (ORQ), Personal Stress Questionnaire (PSQ), and Personal Resources Questionnaire (PRQ). The ORQ consists of 60 questions from 6 dimensions, including occupational overload, role insufficiency, role ambiguity, role boundaries, role responsibility, and physical environment. The PSQ comprises 40 entries from 4 aspects, including vocational stress, psychological stress, interpersonal stress, and physical stress. The PRQ consists of 40 items from 4 dimensions: recreation, self-care, social support and radiation/cognitive coping. Each item is scored using the Likert 5-level scoring method, ranging from “negative” to “often” (1–5). In PSQ and ORQ, higher scores represent higher occupational stress. In PRQ, higher scores represent better capability to cope with occupational stress with less tension. The Cronbach’s α coefficient of this questionnaire was 0.966, and the Cronbach’s α coefficients of the three parts were 0.923, 0.869, and 0.957, respectively.

### Job burnout

The burnout level was measured by the Chinese version of the Maslach Burnout Inventory-General Survey (MBI-GS) [30]. The questionnaire consists of 15 items, including 3 subscales: emotional exhaustion (EE), depersonalization (DP), and reduced sense of personal accomplishment (PA). The 22 items are scored between 0 and 6; the composite score of each scale represents a participant’s total score for each dimension. Participants with scores of 0–18,19–26, and > 27 in the EE scale exhibit low, moderate and high levels of EE, respectively. Those scoring 0–5,6–9, and > 9 in the DP dimension have low, moderate and high levels of DP, respectively. Items in the dimension measuring PA are reverse coded; participants with scores of 0–33,34–39, and > 40 exhibits a low, moderate, and high degree of PA, respectively. Job burnout score =[0.4×EE + 0.3×DP + 0.3 × (6-PA)]. A score < 1.5 indicates that the subject does not have job burnout, 1.5–3.5 indicates that the subject has mild to moderate burnout, and ≥ 3.5 indicates that the subject has severe burnout [31]. The Cronbach’s α coefficient of this questionnaire was 0.910, and the Cronbach’s α coefficients of the three parts were 0.889, 0.868, and 0.863, respectively.

### Depressive symptoms

The mental health status was investigated with the Symptom Checklist 90 (SCL-90), which included 90 items and 10 factors, somatization, obsessive-compulsive symptoms, interpersonal sensitivity, depressive symptoms, anxiety, hostility, phobia, paranoid ideation, psychoticism, and additional item. Each item is scored on a 5-point scale, from “none” to “severe”. A total score of over 160 in SCL-90 denotes the presence of mental health problems [32]. The Cronbach’s α coefficient of this questionnaire was 0.983.

### Confounding factors

Sociodemographic covariates: gender, age, marital status, education, length of service and income were assessed and included in the regression model because of their known relationship to depressive symptoms and each facet of social relationships examined [33]. Information on current confounding factors was obtained by questionnaires.

### Statistical analysis

Questionnaire data were entered into Epidata 3.1 and analyzed using SPSS26.0. Categorical data were expressed as ratios, and intergroup comparisons were analyzed using the χ^2^ test. Unqualified data were analyzed using the Fisher exact probability method. In addition, linear hierarchical regression analysis was conducted to analyze the interaction between job stress and job burnout on the depressive symptoms scores. Bonferroni correction was applied as a multiple testing correction. Furthermore, PROCESS version 3.4.1 in SPSS was used to examine the moderating effects of occupational stress and job burnout, and the significant interactive effects were analyzed using simple slope analysis, after adjusting for gender, age, marital status, education, length of service and income [34]. All reported *P* values were two-tailed, and those less than 0.05 were considered statistically significant.

## Results

### Comparisons between demographic characteristics

We divide individuals into groups based on whether they have encountered occupational stress, the presence and degree of job burnout, and the development of depressive symptoms, using the differential characteristics of scale assessment scores. This classification enables us to analyze and compare the frequency distributions among these various groups. The results showed that the incidence rates of occupational stress, job burnout, and depressive symptoms were 50.58%, 93.47%, and 11.19%, respectively. The results revealed that age, marital status, educational level, length of service, and income all have varying degrees of influence on occupational stress, job burnout, and depressive symptoms (*P* < 0.05). Among these factors, age, length of service and income had a more pronounced impact. Gender did not exhibit a statistically significant difference in its impact on these three factors (Table 1).

**Table 1:** Demographic characteristics and score grades of occupational stress, job burnout and depressive symptoms [N(%)]

Variables	Total	Occupational stress		Job burnout			Depressive symptoms	
		No	Yes	No	Low	High	No	Yes
Gender								
Male	700 (81.59)	337 (39.28)	363 (42.31)	49 (5.71)	139 (16.20)	512 (59.67)	625 (72.84)	75 (8.74)
Female	158 (18.41)	87 (10.14)	71 (8.28)	7 (0.82)	29 (3.38)	122 (14.22)	137 (15.97)	21 (2.45)
Total	858 (100)	424 (49.42)	434 (50.58)	56 (6.53)	168 (19.58)	634 (73.89)	762 (88.81)	96 (11.19)
*χ²*		2.47		1.74			0.861	
*P*		0.116		0.419			0.353	
**Age(years)**								
≤ 25	326 (38.00)	129 (15.03)	197 (22.96)	43 (5.01)	98 (11.42)	185 (21.56)	312 (36.36)	14 (1.63)
25–35	173 (20.16)	78 (9.09)	95 (11.07)	6 (0.70)	24 (2.80)	143 (16.67)	157 (18.30)	16 (1.86)
35–45	217 (25.29)	125 (14.57)	92 (10.72)	2 (0.23)	30 (3.50)	185 (21.56)	180 (20.98)	37 (4.31)
> 45	142 (16.55)	92 (10.72)	50 (5.83)	5 (0.58)	16 (1.86)	121 (14.10)	113 (13.17)	29 (3.38)
Total	858 (100)	424 (49.42)	434 (50.58)	56 (6.53)	168 (19.58)	634 (73.89)	762 (88.81)	96 (11.19)
*χ²*		33.18		87.94			35.94	
*P*		**<0.001**		**<0.001**			**<0.001**	
**Marital status**								
Married	471 (54.90)	273 (31.82)	198 (23.08)	13 (1.52)	70 (8.16)	388 (45.22)	399 (46.50)	72 (8.39)
Unmarried/Divorced/Widowed	387 (45.10)	151 (17.60)	236 (27.51)	43 (5.01)	98 (11.42)	246 (28.67)	363 (42.31)	24 (2.80)
Total	858 (100)	424 (49.42)	434 (50.58)	56 (6.53)	168 (19.58)	634 (73.89)	762 (88.81)	96 (11.19)
*χ²*		30.50		44.75			17.65	
*P*		**<0.001**		**<0.001**			**<0.001**	
**Education**								
Senior high school and below	256 (29.84)	152 (17.72)	104 (12.12)	8 (0.93)	49 (5.71)	199 (23.19)	228 (26.57)	28 (3.26)
Junior school, technical secondary school	471 (54.9)	223 (25.99)	248 (28.9)	42 (4.9)	100 (11.66)	329 (38.34)	416 (48.48)	55 (6.41)
Undergraduate	114 (13.29)	39 (4.55)	75 (8.74)	6 (0.70)	14 (1.63)	94 (10.96)	103 (12.00)	11 (1.28)
Master and above	17 (1.98)	10 (1.17)	7 (0.82)	0 (0)	5 (0.58)	12 (1.40)	15 (1.75)	2 (0.23)
Total	858 (100)	424 (49.42)	434 (50.58)	56 (6.53)	168 (19.58)	634 (73.89)	762 (88.81)	96 (11.19)
*χ²*		22.11		17.38			0.41	
*P*		**<0.001**		**0.008**			0.939	
**Length of service (years)**								
≤ 5	441 (51.40)	188 (21.91)	253 (29.49)	44 (5.13)	113 (13.17)	284 (33.10)	417 (48.6)	24 (2.80)
5–10	67 (7.81)	339 (3.85)	34 (3.96)	2 (0.23)	19 (2.21)	46 (5.36)	64 (7.46)	3 (0.35)
10–20	143 (16.67)	78 (9.09)	65 (7.58)	3 (0.35)	9 (1.05)	131 (15.27)	120 (13.99)	23 (2.68)
> 20	207 (24.13)	125 (14.57)	82 (9.56)	7 (0.82)	27 (3.15)	173 (20.16)	161 (18.76)	46 (5.36)
Total	858 (100)	424 (49.42)	434 (50.58)	56 (6.53)	168 (19.58)	634 (73.89)	762 (88.81)	96 (11.19)
*χ²*		19.60		59.37			46.50	
*P*		**<0.001**		**<0.001**			**<0.001**	
**Income (yuan)**								
≤ 1000	87 (10.14)	47 (5.48)	40 (4.66)	6 (0.7)	16 (1.86)	65 (7.58)	75 (8.74)	12 (1.40)
1000–1500	134 (15.62)	69 (8.04)	65 (7.58)	12 (1.4)	21 (2.45)	101 (11.77)	121 (14.10)	13 (1.52)
1500–2000	307 (35.78)	126 (14.69)	181 (21.10)	32 (3.73)	88 (10.26)	187 (21.79)	289 (33.69)	18 (2.10)
2000–2500	189 (22.03)	106 (12.35)	83 (9.67)	4 (0.47)	21 (2.45)	164 (19.11)	161 (18.76)	28 (3.26)
2500–3000	141 (16.43)	76 (8.86)	65 (7.58)	2 (0.23)	22 (2.56)	117 (13.64)	116 (13.52)	25 (2.91)
Total	858 (100)	424 (49.42)	434 (50.58)	56 (6.53)	168 (19.58)	634 (73.89)	762 (88.81)	96 (11.19)
*χ²*		14.75		57.90			18.26	
*P*		**0.011**		**<0.001**			**0.003**	

### Regression analysis of the effects of occupational stress and job burnout on depressive symptoms

After adjusting for confounding factors, depressive symptoms was regarded as the dependent variable, and subscales of occupational stress and job burnout were considered independent variables. The logistic regression analysis showed that PRQ and PSQ in the occupational stress questionnaires affected depressive symptoms (*P* < 0.05). PRQ was a protective factor with an *OR* (95% *CI*) of 0.96 (0.95, 0.98). PSQ was a risk factor with an *OR* (95% *CI*) of 1.11 (1.09, 1.14). The PA in job burnout is a protective factor against depressive symptoms, with an *OR* (95% *CI*) of 0.92 (0.85, 1.00). On the contrary, EE (*OR* [95% *CI*], 1.36 (1.26, 1.46)) and DP (*OR* [95% *CI*], 1.11 (1.02, 1.21)) were risk factors for depressive symptoms (*P* < 0.05) (Table 2).

**Table 2:** Logistic regression analysis of the factors affecting depressive symptoms

Variables	b	χ²	OR (95%CI)	*P*-vaule
OSI-*R*				
ORQ	-0.01	0.78	0.99 (0.98, 1.01)	0.38
PSQ	0.11	96.46	1.11 (1.09, 1.14)	**< 0.05**
PRQ	-0.04	31.75	0.96 (0.95, 0.98)	**< 0.05**
**CMBI**				
EE	0.30	68.69	1.36 (1.26, 1.46)	**< 0.05**
DP	0.10	5.30	1.11 (1.02, 1.21)	**< 0.05**
PA	-0.09	4.23	0.92 (0.85, 1.00)	**< 0.05**

*Note OR*, odds ratio, *95%CI*, 95% confidence interval. Adjusted for gender, age, marital status, education, length of service and income. Statistically significant values were denoted in bold

### The interactive effects of occupational stress and job burnout on depressive symptoms

The interaction between occupational stress and job burnout was analyzed by linear hierarchical regression analysis (Table 3). As shown in Model 2, the effects of occupational stress and burnout on depressive symptoms (*β* = 0.288, *P* < 0.01, Bonferroni corrected *P* < 0.01; *β* = 0.212, *P* < 0.01, Bonferroni corrected *P* < 0.01, respectively) remained significant after controlling for covariates. In addition, Model 3 showed a significant interaction between occupational stress and job burnout on depressive symptoms (*β* = 0.485, *P* = 0.015, Bonferroni corrected *P* < 0.01). These results suggest that job burnout may be considered as a moderator when examining the association between occupational stress and depressive symptoms in this study.

**Fig. 1:**
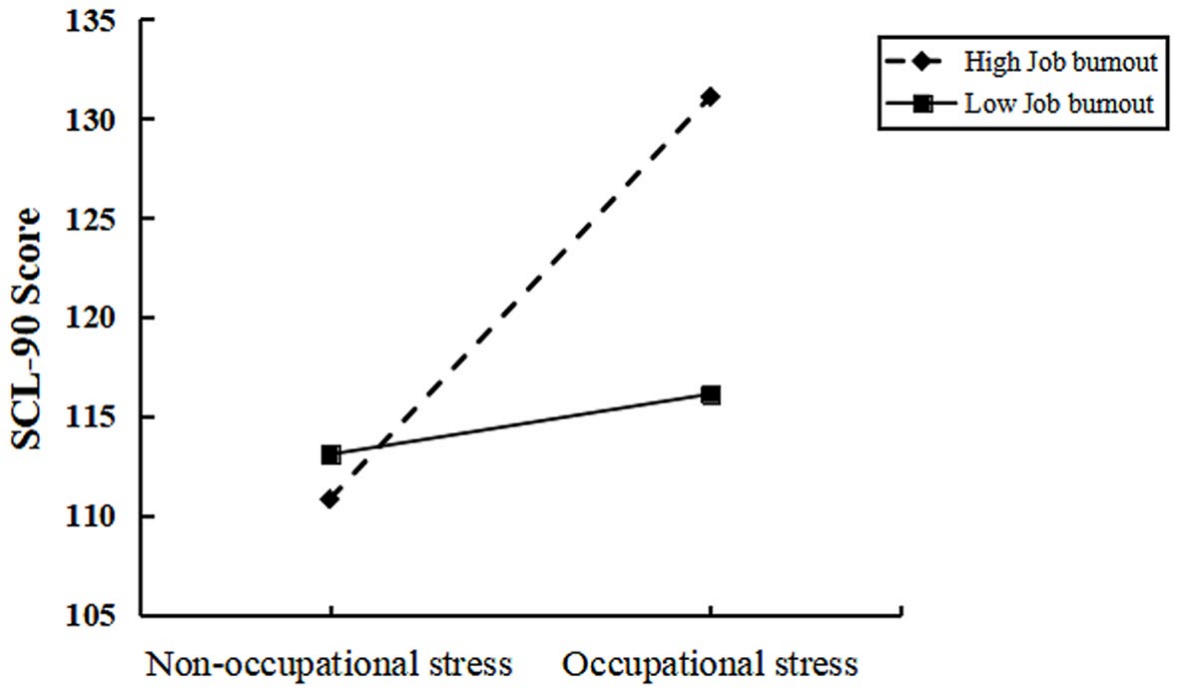
Interaction between occupational stress and job burnout on depressive symptoms

To further explain the moderating role of job burnout between occupational stress and depressive symptom, we analyzed simple slopes within groups with different degrees of burnout after controlling for covariates. The results showed that occupational stress was a significant positive predictor of depressive symptoms at different degrees of burnout. As shown in Fig. 1, suggests that when occupational stress is higher, different degrees of burnout have a risk effect on depression. Even more importantly, the higher the level of job burnout, the greater the risk for depression.

**Table 3:** Multiple moderated regression results (*N* = 858)

Variables	Model1	Model2	Model3
D1 (Female)	0.032	0.039	0.039
D2 (Aged 25–35 years)	**0.117** ^*^	0.059	0.056
D3 (Aged 35–45 years)	0.126	0.078	0.078
D4 (Aged > 45 years)	0.084	0.047	0.047
D5 (Married)	-0.092	-0.055	-0.049
D6 (Senior high school)	**0.084** ^*^	0.07	0.069
D7 (Undergraduate)	0.026	-0.013	-0.014
D8 (Master and above)	0.002	0.01	0.013
D9 (Length of service 5–10 years)	0.007	0.018	0.017
D10 (Length of service 10–20 years)	**0.133** ^**^	**0.112** ^*^	**0.112** ^*^
D11 (Length of service > 20 years)	**0.275** ^**^	**0.261** ^**^	**0.258** ^**^
D12 (Income 1000–1500 yuan)	0.012	0.008	0.004
D13 (Income 1500–2000 yuan)	0.014	0.013	0.012
D14 (Income 2000–2500 yuan)	0.017	0.004	0.002
D15 (Income 2500–3000 yuan)	-0.013	-0.014	-0.017
△R^2^	**0.081** ^**^		
Low Job burnout		0.085	-0.176
High Job burnout		**0.288** ^**^	-0.224
Occupational stress		**0.212** ^**^	-0.229
△R^2^		**0.066** ^**^	
Job burnout × Occupational stress			**0.485** ^*^
△R^2^			**0.006** ^*^

*Note* Linear hierarchical regression was used to examine the moderating effects of occupational stress and job burnout. Depressive symptoms were dependent variable. Model1 included demographic covariates (gender, age, marital status, education, length of service and income), and added the main factors and interactions in model 2 and model 3, respectively. D1-15 were dummy variables. Job stress were standardized variables. Statistically significant values were denoted in bold. *P < 0.05; **P < 0.01

## Discussion

The results of this study found that the incidence of occupational stress among railway workers in Fuzhou City was 50.58%, which was lower than that of employees of medical staff (59.4%) [35] and higher than coal miners (38.1%) [36]. The higher occupational stress scores in the age < 25 years and working experience < 5 years group are consistent with the findings of Ines et al. [37]. It may since this group of workers are new to their jobs, have heavy workloads, are inexperienced, and encounter more difficulties and challenges [38]. The incidence of job burnout among participants was 93.47%, which was higher than that of caregivers (67.37%) [39] and coal miners (77.74%) [36]. The higher burnout scores in the age group of 35–45 years old and 10–20 years of service are consistent with the results of Xian et al. [40], which may related to the fact that this group of workers has been in the job for a long time, but their career development is different from what was envisioned, and their professional enthusiasm diminishes due to a lower sense of professional identity. Furthermore, 11.19% of the participants were defined as depressed and the tendency to depression increased with age and working age, which is consistent with the results reported by Seo JW et al. [41]. The possible explanation for this is that with increasing age and working age, employees’ physical functioning gradually declines, lack of vigor at work, and quality of life decreases, which tends to lead to low mood, thus increasing the chances of depression [42].

The regression analysis indicated that occupational stress and burnout were both risk factors for depressive symptoms. Consistent with certain studies showed that PSQ of occupational stress was a risk factor for depressive symptoms, and PRQ was a protective factor [43]. With the advent of EE, DP, and RA, the degree of job burnout increased with higher odds of developing depressive symptoms, consistent with Alan et al.‘s study [44]. Occupational stress played a significant role in depressive symptoms, and EE induced by job burnout was highly correlated with depressive symptoms [45, 46]. When railway workers face heavy vocational tasks and give more than they get, they will probably be in a state of tension [47]. Stress alters hormone secretion, affecting individuals’ mentality and behavior, leading to EE, DP, and even depressive symptoms [48].

Prolonged occupational stress exposure may lead to persistent feelings of fatigue and exhaustion, which may ultimately develop into job burnout. Among the core dimensions of burnout, emotional exhaustion is strongly associated with depressive symptoms. Research findings have shown that as the level of burnout increases, so does the likelihood of depression, a pattern that has been demonstrated in a variety of occupational groups such as doctors and teachers [49]. Maslach et al.‘s [31] research particularly emphasizes that the interaction and impact of EE, DP, and reduced personal accomplishment (rPA) on one another are crucial in understanding and identifying potential risks for depression when assessing job burnout.

In addition, the potential role of the interaction between occupational stress and job burnout on depression may unknown. Therefore, this study aimed to investigate the interaction between occupational stress and job burnout in the depressive symptoms of Chinese railroad workers and to test whether different levels of job burnout exacerbate the effect of occupational stress on depressive symptoms. First, the interaction between occupational stress and job burnout had a significant effect on depressive symptoms after controlling for covariates. In addition, our study found that subjects with high levels of job burnout scored higher on depressive symptoms under high job stress. Taken together, these results suggest that occupational stress and job burnout interactions influence depressive symptoms, which is supported in several related studies [23, 25]. It is generally understood that under the prolonged influence of occupational stress, if an individual fails to obtain sufficient resources and social support to address the issues, it can potentially lead to the emergence of job burnout [27]. This denotes that there may be an interaction between occupational stress and job burnout, affecting depressive symptoms individually or in combination, consistent with Lin et al.‘s findings [49]. Furthermore, workers under prolonged occupational stress and emotional exhaustion find it challenging to recover from the situation with insufficient resources, thereby affecting physical and mental health [50].

This study suggests that the railway management layer needs to pay more attention to employees’ occupational stress and depressive symptoms. Moreover, heavy occupational tasks, high living stress, occupational identity, and disease status are critical causes of job burnout [15]. Managers in the railway bureau should provide prompt psychological counseling for employees, give them adequate autonomy and control, and raise wages to decrease the occupational stress response and emotional exhaustion. Meanwhile, psychological lectures and vocational training should be provided to foster personal resilience, prevent depressive symptoms, and improve the quality of life of railway workers [51].

Studies may shown that people with high occupational stress are more likely to suffer from depression [52]. The strength of this study is that we developed an interaction model to explore the relationship between occupational stress, job burnout, and depressive symptoms. Nonetheless, this study had some limitations: (1) Our sample represents a population of railway workers in Fuzhou. It is unclear whether the interaction between occupational stress and burnout in depressive symptoms extends to other occupational groups. (2) Assessments of occupational stress, job burnout, and depressive symptoms are based exclusively on subjective questionnaires, which are prone to false-positive results and may affect the accuracy of the results. For example, the SCL-90 can investigate and reflect mental health problems in different occupational groups, but a diagnosis of depression needs to be confirmed on the basis of a clinician’s diagnosis. (3) This study was a cross-sectional study could not establish a causal relationship. Therefore, a longitudinal study is necessary to explore the relationship between occupational stress, job burnout and depressive symptoms.

## Conclusion

Our results found that the depressive symptoms of railway workers in Fuzhou city was related to occupational stress and job burnout. The higher ORQ and PSQ of occupational stress, the higher the possibility of depressive symptoms was. The more serious the EE, DP and PA of job burnout are, the more obvious the depressive tendency will be. More importantly, occupational stress and varying degrees of burnout interact to influence depressive symptoms, especially under high job stress and high burnout. These findings laid a foundation for studying the correlation between occupational stress and depressive symptoms and the impact of job burnout on the mental health of workers. For workers, the adjustment of the neglected job burnout may be an effective way to reduce depressive symptoms at work life.

## Data Availability

The datasets analyzed in the current study are available from the corresponding author on reasonable request.

## Abbreviations

OSI-R: Occupational Stress Inventory Revised Edition
CMBI: China Job Burnout Inventory
SCL-90: Symptom Checklist-90
ORQ: Occupational Roles Questionnaire
PSQ: Personal Stress Questionnaire
PRQ: Personal Resources Questionnaire
MBI-GS: Maslach Burnout Inventory-General Survey
EE: Emotional exhaustion
DP: Depersonalization
PA: Personal accomplishment

## References

[1] Pikhart H (2017). Johannes Siegrist and Morten Wahrendorf, editors. Work Stress and Health in a globalized economy. The model of effort-reward imbalance. Eur J Pub Health.

[2] Li X, Gao X, Liu J. Cross-sectional survey on the relationship between occupational stress, hormone levels, and the Sleep Quality of Oilfield Workers in Xinjiang, China. Int J Environ Res Public Health 2019, 16(18). 10.3390/ijerph16183316 PMC6765891 31505823

[3] Girma B, Nigussie J, Molla A, Mareg M (2021). Occupational stress and associated factors among health care professionals in Ethiopia: a systematic review and meta-analysis. BMC Public Health.

[4] Zhang M, Murphy B, Cabanilla A, Yidi C (2021). Physical relaxation for occupational stress in healthcare workers: a systematic review and network meta-analysis of randomized controlled trials. J Occup Health.

[5] Restrepo J, Lemos M (2021). Addressing psychosocial work-related stress interventions: a systematic review. Work.

[6] Milner A, Scovelle AJ, King TL, Madsen I (2019). Exposure to work stress and use of psychotropic medications: a systematic review and meta-analysis. J Epidemiol Community Health.

[7] Taouk Y, Spittal MJ, LaMontagne AD, Milner AJ (2020). Psychosocial work stressors and risk of all-cause and coronary heart disease mortality: a systematic review and meta-analysis. Scand J Work Environ Health.

[8] Gu B, Tan Q, Zhao S (2019). The association between occupational stress and psychosomatic wellbeing among Chinese nurses: a cross-sectional survey. Med (Baltim).

[9] Serrão C, Duarte I, Castro L, Teixeira A. Burnout and depression in Portuguese Healthcare Workers during the COVID-19 pandemic-the mediating role of psychological resilience. Int J Environ Res Public Health 2021, 18(2). 10.3390/ijerph18020636 PMC7828555 33451083

[10] Ramírez-Elvira S, Romero-Béjar JL, Suleiman-Martos N, Gómez-Urquiza JL, Monsalve-Reyes C, Cañadas-De la Fuente GA, Albendín-García L. Prevalence, Risk Factors and Burnout Levels in Intensive Care Unit Nurses: A Systematic Review and Meta-Analysis. Int J Environ Res Public Health 2021, 18(21). 10.3390/ijerph182111432 PMC8583312 34769948

[11] Bagaajav A, Myagmarjav S, Nanjid K, Otgon S, Chae YM (2011). Burnout and job stress among Mongolian doctors and nurses. Ind Health.

[12] Yörük S, Güler D (2021). The relationship between psychological resilience, burnout, stress, and sociodemographic factors with depression in nurses and midwives during the COVID-19 pandemic: a cross-sectional study in Turkey. Perspect Psychiatr Care.

[13] Çakıcı M, Gökçe Ö, Babayiğit A, Çakıcı E, Eş A (2017). Depression: point-prevalence and risk factors in a North Cyprus household adult cross-sectional study. BMC Psychiatry.

[14] Madsen IEH, Nyberg ST, Magnusson Hanson LL, Ferrie JE, Ahola K, Alfredsson L, Batty GD, Bjorner JB, Borritz M, Burr H (2017). Job strain as a risk factor for clinical depression: systematic review and meta-analysis with additional individual participant data. Psychol Med.

[15] Agrawal V, Plantinga L, Abdel-Kader K, Pivert K, Provenzano A, Soman S, Choi MJ, Jaar BG (2020). Burnout and Emotional Well-Being among Nephrology fellows: a National Online Survey. J Am Soc Nephrol.

[16] Malhi GS, Mann JJ (2018). Depress Lancet.

[17] Liu Q, He H, Yang J, Feng X, Zhao F, Lyu J (2020). Changes in the global burden of depression from 1990 to 2017: findings from the Global Burden of Disease study. J Psychiatr Res.

[18] Chen C, Meier ST (2021). Burnout and depression in nurses: a systematic review and meta-analysis. Int J Nurs Stud.

[19] Cañadas-de la Fuente GA, Albendín-García L, San Luis-Costas GRC, de la Ortega-Campos C (2018). Fuente-Solana EI: nurse burnout in critical care units and emergency departments: intensity and associated factors. Emergencias.

[20] Hu TQ, Chen ZB, Liu W, Jiang Y (2021). [Effect of occupational stress on the mental health of railway workers]. Zhonghua Lao Dong Wei Sheng Zhi Ye Bing Za Zhi.

[21] Jiang Y, Wu C, Hu T, Chen M, Liu W, Zhou Y, Chen Z, Xu X (2020). Association for combined exposure to job strain, shift work on mental health among Chinese railway workers: a cross-sectional study. BMJ Open.

[22] Tàpia-Caballero P, Serrano-Fernández MJ, Boada-Cuerva M, Araya-Castillo L, Boada-Grau J (2022). Variables that predict burnout in professional drivers. Int J Occup Saf Ergon.

[23] Hsieh HF, Liu Y, Hsu HT, Ma SC, Wang HH, Ko CH. Relations between stress and depressive symptoms in Psychiatric nurses: the Mediating effects of Sleep Quality and Occupational Burnout. Int J Environ Res Public Health 2021, 18(14). 10.3390/ijerph18147327 PMC8303432 34299778

[24] Wang Y, Zhao M, Li P, Wu C, Lv Y, Jiang Y (2022). Gene-environment interaction between circadian clock gene polymorphisms and job stress on the risk of sleep disturbances. Psychopharmacology.

[25] Gu H, Lee J, Hwang Y, Kim J, Lee S, Kim SJ (2023). Job burnout among workers with different shift regularity: interactive factors between sleep, depression, and work environment. Front Public Health.

[26] Lee S, Lee J, Jeon S, Hwang Y, Kim J, Kim SJ (2023). Sleep disturbances and depressive symptoms of shift workers: effects of shift schedules. J Psychiatr Res.

[27] Koutsimani P, Montgomery A, Georganta K (2019). The Relationship between Burnout, Depression, and anxiety: a systematic review and Meta-analysis. Front Psychol.

[28] Xu W, Sun H, Zhu B, Yu X, Niu Y, Kou C, Li W (2021). The prevalence of depressive symptoms and its determinants among adults in mainland China: results from a national household survey. J Affect Disord.

[29] Quan L, Zhang Y, Jiang F, Liu Y, Lan Y, Huang L (2022). Influence of workload, personality, and psychological flexibility on occupational stress among Medical Staff: a fuzzy-set qualitative comparative analysis. Front Public Health.

[30] Li X, Jiang T, Sun J, Shi L, Liu J (2021). The relationship between occupational stress, job burnout and quality of life among surgical nurses in Xinjiang, China. BMC Nurs.

[31] Maslach C, Jackson SE (1981). The measurement of experienced burnout. J Organizational Behav.

[32] Schreuder MJ, Hartman CA, George SV, Menne-Lothmann C, Decoster J, van Winkel R, Delespaul P, De Hert M, Derom C, Thiery E, Rutten BPF, Jacobs N, van Os J, Wigman JTW, Wichers M (2020). Early warning signals in psychopathology: what do they tell?. BMC Med.

[33] Werneck AO, Vancampfort D, Stubbs B, Silva DR, Cucato GG, Christofaro DGD, Santos RD, Ritti-Dias RM, Bittencourt MS (2022). Prospective associations between multiple lifestyle behaviors and depressive symptoms. J Affect Disord.

[34] Hayes AF. PROCESS: A Versatile Computational Tool for Observed Variable Mediation, Moderation, and Conditional Process Modeling 1. In: 2012; 2012.

[35] Zhu D, Wang J, Zhao Y, Yang L, Gao J, Chang X, Li S, Zheng Y. The status of occupational stress and its influence on the Health of Medical Staff in Lanzhou, China. Int J Environ Res Public Health 2022, 19(17). 10.3390/ijerph191710808 PMC9518311 36078517

[36] Fu A, Zhao T, Gao X, Li X, Liu X, Liu J (2022). Association of psychological symptoms with job burnout and occupational stress among coal miners in Xinjiang, China: a cross-sectional study. Front Public Health.

[37] Carmona-Barrientos I, Gala-León FJ, Lupiani-Giménez M, Cruz-Barrientos A, Lucena-Anton D, Moral-Munoz JA (2020). Occupational stress and burnout among physiotherapists: a cross-sectional survey in Cadiz (Spain). Hum Resour Health.

[38] Hege A, Lemke MK, Apostolopoulos Y, Sönmez S (2019). The impact of Work Organization, job stress, and sleep on the Health behaviors and outcomes of U.S. Long-Haul Truck drivers. Health Educ Behav.

[39] Liu L, Tian HE, Wang Y, Xu SH, Jia SF, Zhang L, Zhou LP, Tian JH (2020). [The current situation of occupational burnout and its influencing factors among orphan child care workers in Nanjing]. Zhonghua Lao Dong Wei Sheng Zhi Ye Bing Za Zhi.

[40] Yong X, Gao X, Zhang Z, Ge H, Sun X, Ma X, Liu J (2020). Associations of occupational stress with job burn-out, depression and hypertension in coal miners of Xinjiang, China: a cross-sectional study. BMJ Open.

[41] Seo JW, Lee J, Jeon S, Hwang Y, Kim J, Lee S, Kim SJ (2023). Fatigue and somatization in shift-workers: effects of depression and sleep. J Psychosom Res.

[42] Moreno Fortes A, Tian L, Huebner ES. Occupational Stress and Employees Complete Mental Health: a cross-cultural empirical study. Int J Environ Res Public Health 2020, 17(10). 10.3390/ijerph17103629 PMC7277686 32455763

[43] Schonfeld IS, Bianchi R (2021). From burnout to Occupational Depression: recent developments in Research on Job-related distress and Occupational Health. Front Public Health.

[44] Wang YW, Liu GZ, Zhou XT, Sheng PJ, Cui FF, Shi T (2017). [Mediating effect of mental elasticity on occupational stress and depression in female nurses]. Zhonghua Lao Dong Wei Sheng Zhi Ye Bing Za Zhi.

[45] Oliveira AM, Silva MT, Galvão TF, Lopes LC (2018). The relationship between job satisfaction, burnout syndrome and depressive symptoms: an analysis of professionals in a teaching hospital in Brazil. Med (Baltim).

[46] Parker G, Tavella G (2021). Distinguishing burnout from clinical depression: a theoretical differentiation template. J Affect Disord.

[47] Ren MX, Tian HE, Ma L, Zhou LP, Wang Y (2018). [Comparison of occupational stress and its factors of workers in an Oil Refinery]. Zhonghua Lao Dong Wei Sheng Zhi Ye Bing Za Zhi.

[48] Lu Y, Li Z, Fan Y, Wang J, Zhong T, Wang L, Xiao Y, Zhang D, Chen Q, Yu X. The mediating role of cumulative fatigue on the Association between Occupational Stress and depressive symptoms: a cross-sectional study among 1327 Chinese primary Healthcare professionals. Int J Environ Res Public Health 2022, 19(23). 10.3390/ijerph192315477 PMC9735977 36497554

[49] Lin TC, Lin HS, Cheng SF, Wu LM, Ou-Yang MC (2016). Work stress, occupational burnout and depression levels: a clinical study of paediatric intensive care unit nurses in Taiwan. J Clin Nurs.

[50] Akova İ, Kiliç E, Özdemir ME (2022). Prevalence of Burnout, Depression, anxiety, stress, and Hopelessness among Healthcare Workers in COVID-19 pandemic in Turkey. Inquiry.

[51] Pereira H, Feher G, Tibold A, Costa V, Monteiro S, Esgalhado G. Mediating effect of Burnout on the Association between Work-Related Quality of Life and Mental Health Symptoms. Brain Sci 2021, 11(6). 10.3390/brainsci11060813 PMC8235172 34205291

[52] Liu L, Chang Y, Fu J, Wang J, Wang L (2012). The mediating role of psychological capital on the association between occupational stress and depressive symptoms among Chinese physicians: a cross-sectional study. BMC Public Health.

